# Stochastic Expression of Sae-Dependent Virulence Genes during Staphylococcus aureus Biofilm Development Is Dependent on SaeS

**DOI:** 10.1128/mBio.03081-19

**Published:** 2020-01-14

**Authors:** Elizabeth A. DelMain, Derek E. Moormeier, Jennifer L. Endres, Rebecca E. Hodges, Marat R. Sadykov, Alexander R. Horswill, Kenneth W. Bayles

**Affiliations:** a Department of Pathology and Microbiology, University of Nebraska Medical Center, Omaha, Nebraska, USA; b Department of Immunology and Microbiology, University of Colorado School of Medicine, Aurora, Colorado, USA; KUMC

**Keywords:** *Staphylococcus aureus*, biofilms, developmental biology, gene regulation, stochastic expression, virulence factors

## Abstract

The intricate process of biofilm formation in the human pathogen Staphylococcus aureus involves distinct stages during which a complex mixture of matrix molecules is produced and modified throughout the developmental cycle. Early in biofilm development, a subpopulation of cells detaches from its substrate in an event termed “exodus” that is mediated by SaePQRS-dependent stochastic expression of a secreted staphylococcal nuclease, which degrades extracellular DNA within the matrix, causing the release of cells and subsequently allowing for the formation of metabolically heterogenous microcolonies. Since the SaePQRS regulatory system is involved in the transcriptional control of multiple S. aureus virulence factors, the expression of several additional virulence genes was examined within a developing biofilm by introducing fluorescent gene reporter plasmids into wild-type S. aureus and isogenic regulatory mutants and growing these strains in a microfluidic system that supplies the bacteria with a constant flow of media while simultaneously imaging developing biofilms in 5-min intervals. This study demonstrated that multiple virulence genes, including *nuc*, were expressed stochastically within a specialized subpopulation of cells in nascent biofilms. We demonstrated that virulence genes regulated by SaePQRS were stochastically expressed in nearly all strains examined whereas Agr-regulated genes were expressed more homogenously within maturing microcolonies. The commonly used Newman strain contains a variant of SaeS (SaeS^P^) that confers constitutive kinase activity to the protein and caused this strain to lack the stochastic expression pattern observed in other strain backgrounds. Importantly, repair of the SaeS^P^ allele resulting in reversion to the well-conserved SaeS*^L^* allele found in other strains restored stochastic expression in this strain.

## Abstract (precis)

Staphylococcus aureus is an important human pathogen capable of colonizing diverse tissue types and inducing severe disease in both immunocompromised and otherwise healthy individuals. Biofilm infections caused by this bacterial species are of particular concern because of their persistence, even in the face of intensive therapeutic intervention. The results of the current study demonstrate the stochastic nature of Sae-mediated virulence gene expression in S. aureus and indicate that this regulatory system may function as a “bistable switch” in a manner similar to that seen with regulators controlling competence gene expression in Bacillus subtilis and persister cell formation in Escherichia coli. The results of this study provide a new perspective on the complex mechanisms utilized by S. aureus during the establishment of infections.

## INTRODUCTION

Staphylococcus aureus is a medically important pathogen capable of both asymptomatic nasal colonization as well as acute and chronic infections in a wide array of tissue types (1, 2). The versatility of this organism can be attributed to the combination of the vast repertoire of virulence factors it produces, its ability to form biofilm, and its metabolic adaptability (3, 4). The expression of virulence factors and the processes associated with biofilm development involve numerous interacting regulators, including two-component signal transduction systems (Agr and SaePQRS being two well-studied examples), stand-alone transcriptional regulators (such as Rot, SarA, and CodY), and an alternative sigma factor (e.g., σ^B^) (5–7). Studies have shown that the bacterial gene expression profile induced under planktonic growth conditions differs from that observed in cells grown as part of a biofilm (8, 9). Epidemiological and etiological evidence indicates that infections caused by S. aureus commonly involve the formation of biofilms on implanted or indwelling medical devices such as catheters, prosthetic orthopedic joints, and cardiac devices (10–13). Additionally, the host response elicited by a biofilm infection differs from that elicited by planktonic bacteria (14, 15). The majority of what is known about S. aureus regulatory systems has been obtained through studies of planktonic cultures; however, the biofilm mode of growth may represent a more appropriate context to understand the complex interactions between these regulatory systems.

S. aureus biofilm development is a complex process that involves the production of multiple extracellular matrix (ECM) components, including polysaccharide intercellular adhesin (PIA), extracellular DNA (eDNA), fibronectin binding protein (FnBP), and cell wall-anchored clumping factors (ClfA and ClfB), as well as extracellular adherence protein (Eap) (16–18). The complex nature of biofilm development is further illustrated by recent studies that utilized time-lapse microscopy to gain a much more detailed assessment of the different developmental stages that exist and of the gene expression changes that occur during S. aureus biofilm formation. These studies also revealed a previously unrecognized stage of biofilm development, termed “exodus,” that precedes microcolony formation and the subsequent Agr-mediated dispersal phase (19). The results of this study demonstrated that the exodus event was dependent on the stochastic expression of the *nuc* gene, encoding staphylococcal nuclease. The significance of production of this enzyme during biofilm development was highlighted by the demonstration that nuclease production leads to the degradation of the eDNA that functions to maintain the structural stability of the biofilm (20, 21).

In the present study, we continued our assessment of the stochastic nature of *nuc* expression during S. aureus biofilm formation using fluorescent reporter gene-based approaches that allow for monitoring of the transcription changes that occur at the cellular level. The results demonstrate that stochastic *nuc* expression and the exodus stage of biofilm development are wide-spread phenomena, occurring in all strains tested, representative of four major S. aureus clonal complexes. We also show, for the first time, that several other virulence genes, including those encoding staphylocoagulase (*coa*) and staphylococcal enterotoxin-like toxin X (*selX*), exhibit stochastic expression during biofilm development, identifying a specialized subpopulation of cells dedicated to the production of these virulence factors. Furthermore, utilizing isogenic *agr* and *sae* mutants to examine the roles of the Agr and Sae virulence regulatory systems in stochastic gene expression, we demonstrated that, like the exodus stage of biofilm development, stochastic gene expression is independent of Agr quorum sensing and is instead dependent on the activity of the SaeS sensor histidine kinase.

## RESULTS

### Stochastic *nuc* expression and exodus are observed in multiple clonal complexes.

**FIG 1:**
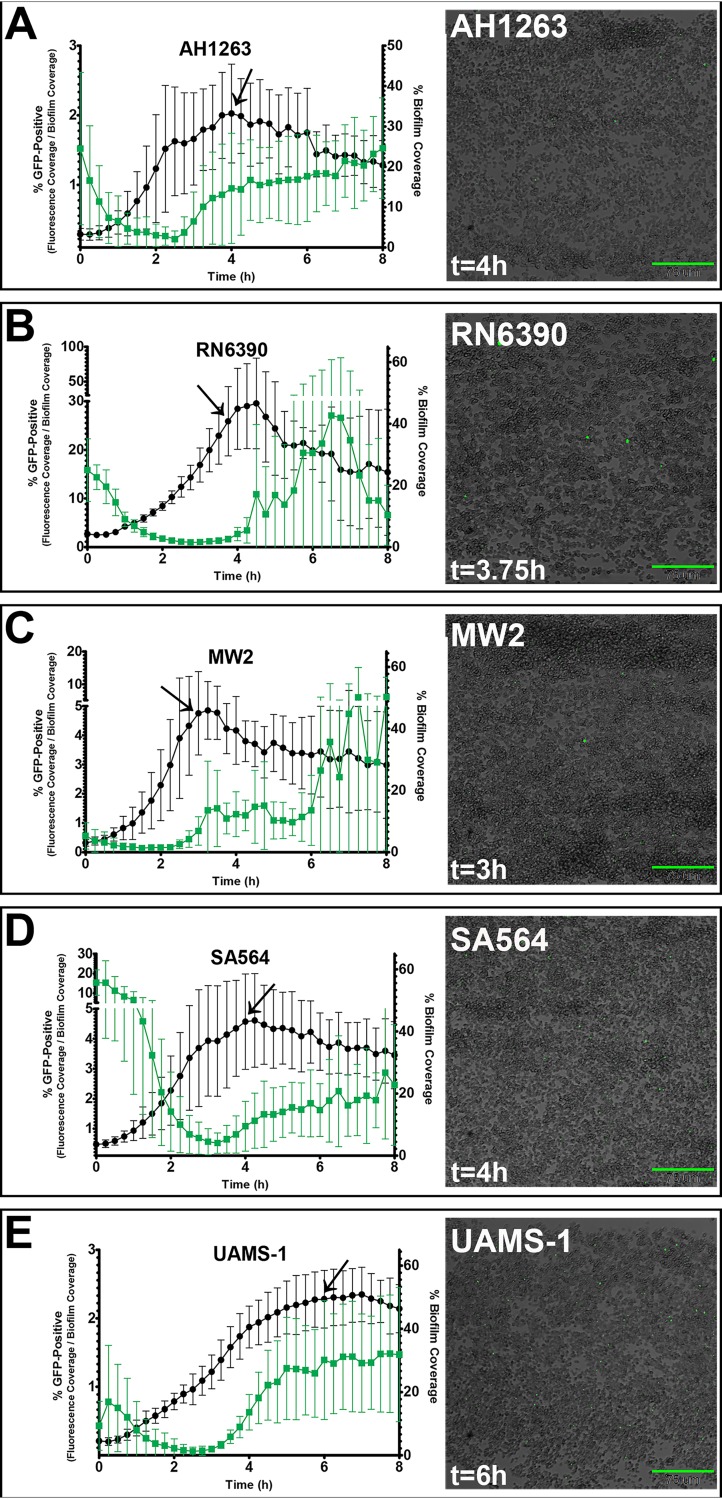
Assessment of exodus and stochastic expression in different genetic backgrounds. S. aureus strains containing the *nuc*::*gfp* reporter plasmid (pCM20) were grown in a BioFlux system, where bright-field and epifluorescent images were acquired at 5-min intervals at ×200 magnification. The images shown are bright-field and epifluorescent overlays that depict individual cells within the biofilm, some of which produce a fluorescent signal. The acquisition time is indicated in the lower left corner of each biofilm image and with an arrow on the corresponding plot; differences in acquisition times reflect the variability in timing associated with exodus. Scale bars represent 75 μm. Each plot depicts the average percentage of channel area covered by cells (represented by black circles and plotted on the right *y* axis) and the average percentage of biofilm area that was GFP positive (represented by green squares and plotted on the left *y* axis) at 15-min intervals over 8 h of growth. The minimum and maximum values of the *y* axes were adjusted in some cases to ensure that the full range of mean values over the entire growing period were visible. The data represent means of results from three independent experiments, each containing at least two technical replicates. Error bars represent standard deviations (SD).

Previous reports from our laboratory revealed that stochastic expression of the staphylococcal nuclease gene (*nuc*) during early biofilm formation induces the newly recognized stage of development, referred to as “exodus,” which precedes the formation of microcolonies during biofilm maturation (19). To determine if exodus occurs in other staphylococcal strains, we examined strains that represented four common S. aureus clonal complexes, including UAMS-1 (CC30), AH1263 and RN6390 (CC8), SA564 (CC5), and MW2 (CC1), using a BioFlux microfluidics system as previously described (19). Biofilms generated by all of these strains showed the same developmental pattern. First, cells replicated and formed a thin “mat,” characteristic of the multiplication stage of development. Multiplication was followed by exodus, at which time a subset of cells detached, leaving behind cells that were randomly dispersed across the channel wall. The remaining cells continued to replicate as they transitioned into the maturation stage of biofilm development, during which microcolony formation occurred. As shown in Fig. 1, the initial accumulation of biomass, corresponding to the multiplication stage of development, occurred over the course of 3 to 7 h, with the durations differing between genetic backgrounds. On average, UAMS-1 (Fig. 1E) took 7 h to reach peak cell accumulation whereas MW2 (Fig. 1C) showed peak cell accumulation as early as 3 h into the experiment. The exodus event followed this peak in cell accumulation and was associated with a decline in cell coverage (Fig. 1). The conclusion of exodus was associated with the initiation of microcolony formation that is a characteristic of the maturation stage of development.

Since exodus occurred in all strains tested, we reasoned that the timing of *nuc* expression would be associated with the exodus event. To test this, we utilized the previously described *nuc* reporter plasmid (pCM20; 21) to monitor *nuc* expression in individual cells during biofilm development. As anticipated, *nuc* expression was observed during biofilm development in all the tested strains and roughly corresponded to the timing of the exodus event (Fig. 1). As was reported previously (19), only a relatively small subpopulation of cells actually generated a fluorescent signal (Fig. 1). In addition to variations in the times at which the different strains underwent the stages of development, the percentage of channel area covered by cells also varied by strain (Fig. 1). In order to normalize for cell density and address the question of whether similar proportions of the populations expressed *nuc* among the strains tested, we quantified the percentage of the population that fluoresced during biofilm development. The results revealed that the percentages of *nuc*-positive cells within the biofilms also differed from strain to strain (Fig. 1). For example, UAMS-1 biofilms, on average, reached peak cell coverage when 50% of the channel area was covered (after 7 h of growth) but only 1% was green fluorescent protein (GFP) positive at that time point (Fig. 1E). In contrast, cell accumulation for RN6390 peaked with an average cell coverage area of 45% (after 4.5 h of growth) but approximately 10% of this population was GFP positive (Fig. 1B). Another example of differences in levels of *nuc* expression between strains can be seen by the high percentage of *nuc*-positive cells in SA564 (Fig. 1D) and RN6390 (Fig. 1B) biofilms at the start of the experiment compared with the relatively low percentage of GFP-positive cells within the biofilms of the other strains at this time point (Fig. 1A, C, and E). After the initiation of the experiment, the percentage of high-expressing cells declined over time but then increased when the stochastic expression of *nuc* resumed (Fig. 1). Although we examined hundreds of images of nascent biofilms to determine if this expression could be explained by the presence of subtle environmental/spatial characteristics (proximity to other cells, the relative position of attachment to the viewing channel, the expression level of neighboring cells, etc.), no evidence of this was found. Thus, we conclude that *nuc* is stochastically expressed by S. aureus during biofilm development and that this mode of expression is common in a variety of strains representing multiple clonal complexes.

### The specific roles of Sae and Agr during biofilm development.

**FIG 2:**
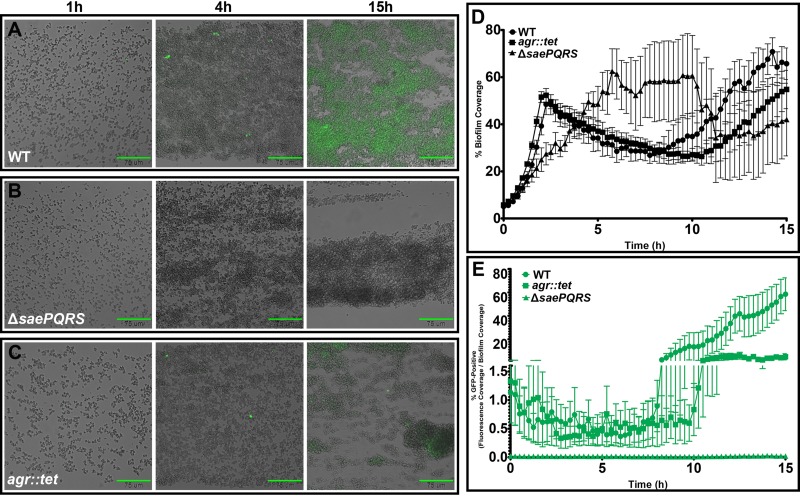
Effect of the Sae and Agr regulatory systems on *nuc* expression during biofilm development. (A to C) The S. aureus wild-type (WT) strain (AH1263) (A) and isogenic mutants Δ*saePQRS* (AH2216) (B) and *agr*::*tet* (AH1292) (C) harboring the *nuc*::*gfp* reporter were grown in a BioFlux system, where bright-field and epifluorescent images were acquired at 5-min intervals at ×200 magnification). The images shown are bright-field and epifluorescent overlays taken at the indicated times during biofilm development. Scale bars represent 75 μm. (D and E) Graphs depict the average percentage of channel area covered by cells (D) and the average percentage of the biofilm area that was GFP positive at 15-min intervals over 15 h of growth (E). The data represent means of results from three independent experiments, each containing at least two technical replicates. Error bars represent the SD. After 5 h of growth, Δ*saePQRS* mutant biofilms often caused the microfluidic channels to clog due to the buildup of cells resulting from deficient nuclease production (19).

It was demonstrated in a previous study that exodus occurred in an Sae-dependent but Agr-independent manner (19). Given that the exodus event is mediated by *nuc* (19), we hypothesized that stochastic expression from the *nuc*::*gfp* reporter would still be observed in an *agr* mutant. Indeed, early stochastic expression of the *nuc* reporter was readily observed in the *agr* mutant (AH1292, Fig. 2C) at levels comparable to those of the parental strain (AH1263, Fig. 2A) whereas it was substantially diminished in the *saePQRS* mutant (AH2216, Fig. 2B). Furthermore, as was observed during exodus, the *saePQRS* mutant biofilm failed to induce fluorescent signal during maturation despite forming cell aggregates that appeared similar in size to those formed by the wild-type and *agr* mutant strains (Fig. 2).

Early reports examining the necessity of Sae and Agr in virulence gene expression found activation of the Sae system to be at least partially dependent on Agr (22, 23). However, more recent reports argued against any Agr dependence in Sae-mediated signaling, particularly in *nuc* expression (24). Quantification of wild-type and *agr* mutant biofilm coverage over time showed that the levels of early biofilm development of the two strains were very similar (Fig. 2D). At early time points (*t* = 0 h to *t* = 8 h), pairwise comparisons found no statistically significant difference in either cell coverage (all adjusted *P* values > 0.15) or the percentage of cells that were fluorescent (all adjusted *P* values > 0.27). It was not until later in biofilm development (after 9 h of growth) that any discernible differences between the two emerged. The *agr* mutant biofilm took slightly longer, on average, to reach maturation, and the percentage of the channel area that was covered by biofilm at later time points was often less than that covered by the wild-type biofilms (Fig. 2D; see also Fig. S1 in the supplemental material). Despite the minimal differences in the area of channel coverage between the two strains (as indicated in Fig. 2D), a smaller population of cells expressed GFP in towers produced by the *agr* mutant (Fig. 2E). Pairwise comparisons of the wild-type and *agr* mutant biofilms indicated no statistically significant difference in the levels of cell coverage at 9 h (adjusted *P* value = 0.3095) or 15 h (adjusted *P* value = 0.3163). However, there was a statistically significant difference in the percentages of cells that were fluorescent at 9 h (adjusted *P* value = 0.009) or 15 h (adjusted *P* value = <0.0001). The observed differences were even more evident at later time points (Fig. S1). Overall, the results generated by these studies provide further evidence that the mechanisms confining expression of *nuc* to a subpopulation of cells during the early stages of biofilm development are independent of the Agr-mediated quorum sensing system but are dependent on Sae. In contrast, *nuc* expression is dependent on both Sae and Agr during the maturation stage of biofilm development.

10.1128/mBio.03081-19.1 FIG S1 Effect of Agr on expression of *nuc* during maturation. S. aureus wild-type (AH1263) (A) and *agr* mutant (AH1292) (B) strains containing a *nuc*::*gfp* reporter plasmid (pDM19) were grown in the BioFlux system; bright-field and epifluorescent images were acquired at 5-min intervals at ×200 magnification. The acquired images were taken after 18 h of growth and are representative of results from three independent experiments, each containing at least two technical replicates of each strain. Scale bars represent 75 μm. The *agr* mutant biofilms frequently had microcolonies which appeared larger than those seen in wild-type biofilms. Despite this, the fluorescent signal within *agr* mutant towers was often diminished compared to the wild-type strain. Download FIG S1, TIF file, 2.9 MB. Copyright © 2020 DelMain et al. 2020 DelMain et al. This content is distributed under the terms of the Creative Commons Attribution 4.0 International license.

### Sae- and Agr-regulated virulence genes display distinct patterns of expression during biofilm development.

**FIG 3:**
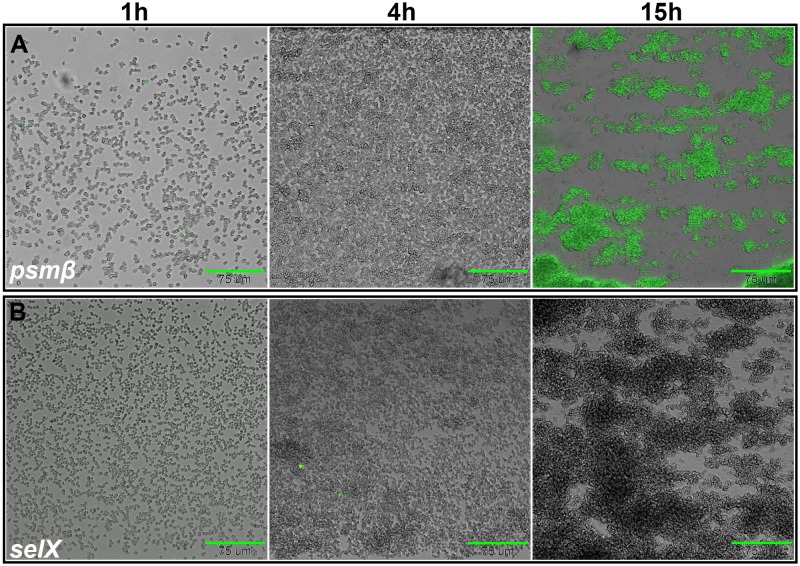
Agr- and Sae-dependent regulation dictate distinct gene expression patterns during biofilm development. The wild-type S. aureus strain (AH1263) harboring the *psm*β reporter (pLD24 (A) and the *selX* reporter (pLD9) (B) were grown in a BioFlux system, where bright-field and epifluorescent images were acquired at 5-min intervals at ×200 magnification. The images shown are bright-field and epifluorescent overlays collected at the indicated times and are representative of results from three independent experiments, each containing at least two technical replicates for each strain. Scale bars represent 75 μm. The Sae-independent virulence gene, *psm*β, is not expressed in a stochastic manner during exodus; instead, Agr-dependent expression occurs in developing towers, during maturation. In contrast, the Sae-dependent virulence gene (*selX*) is stochastically expressed during exodus but signal from this Agr-independent gene is absent from developing towers.

The Agr quorum sensing system becomes activated when sufficient levels of the autoinducing peptide (AIP) are produced and sensed by the sensor histidine kinase, AgrC, a process that occurs within microcolonies during the maturation phase of biofilm development (25). On the basis of our observation that stochastic expression of *nuc* during the exodus stage was independent of the Agr regulatory system, we hypothesized that virulence factor genes regulated by Agr, but independent of Sae, would lack early stochastic expression and that promoter activity for these genes would be limited to the maturation stage when microcolony development occurs. Although there are several genes that fall into this category, the genes encoding the Agr two-component signal transduction system (*agrBCDA*), as well as those encoding the virulence factors β-type phenol-soluble modulins (*psm*β), phosphatidylinositol-specific phospholipase C (*plc*), and staphylococcal enterotoxin K (*sek*), were selected to generate fluorescent promoter fusion reporters. As anticipated, stochastic expression of these genes was not observed during the multiplication or exodus stages of biofilm development (Fig. 3A; see also Fig. S2). In contrast, significant expression of these promoters was observed during the maturation stage, specifically within the developing microcolonies.

10.1128/mBio.03081-19.2 FIG S2 Expression of Sae-independent virulence genes during biofilm development. Cells of the S. aureus wild-type strain (AH1263) containing fluorescent reporter plasmids for virulence genes *plc* (A) and *sek* (B) and the P2 promoter of *agr* (encoding *agrBCDA*) (C) were grown in a BioFlux system, where bright-field and epifluorescent images were acquired at 5-min intervals at ×200 magnification. The images shown are overlays of bright-field and epifluorescent images taken after 4 h (left) and 15 h (right) of growth and are representative of results from three independent experiments, each containing at least two technical replicates of each strain. Scale bars represent 75 μm. Download FIG S2, TIF file, 2.4 MB. Copyright © 2020 DelMain et al. 2020 DelMain et al. This content is distributed under the terms of the Creative Commons Attribution 4.0 International license.

On the basis of the observation that *nuc* is stochastically expressed in a subpopulation of cells in a manner dependent on the SaePQRS system, we hypothesized that other virulence factors under the control of this regulatory system would likewise show similar stochastic expression. To test this, fluorescent promoter fusions were generated for several other virulence genes within the Sae regulon. Stochastic expression was observed for the genes encoding other Sae-dependent virulence genes. Staphylococcal enterotoxin-like toxin X (*selX* [26]) (Fig. 3B) is one such example. Similarly to *nuc* expression, the stochastic expression of *selX* (shown in the context of a dual reporter in Fig. S3) and additional Sae-dependent virulence genes (unpublished results) was observed in the Agr mutant but absent in the Sae mutant during biofilm development. These results indicate that stochastic expression in S. aureus is associated with virulence genes that are regulated by the Sae-regulatory system.

10.1128/mBio.03081-19.3 FIG S3 Coexpression of *nuc* and *selX* is dependent on Sae and independent of Agr during biofilm development. S. aureus regulatory mutant strains Δ*saePQRS* (AH2216) (A) and *agr*::*tet* (AH1292) (B) harboring the *nuc* and *selX* (pLD13) dual reporter were grown in a BioFlux system, where bright-field and epifluorescent images were acquired at 5-min intervals at ×200 magnification. The images shown were taken after 4 h of growth. Scale bars represent 75 μm. Images are representative of results from three independent experiments, each containing at least two technical replicates of each strain. Download FIG S3, TIF file, 1.2 MB. Copyright © 2020 DelMain et al. 2020 DelMain et al. This content is distributed under the terms of the Creative Commons Attribution 4.0 International license.

Collectively, these results indicate that the distinct patterns of expression observed during biofilm development are mediated by Sae- and Agr-dependent regulation. Expression of *selX*, a virulence gene whose expression is Sae-dependent and Agr-independent was observed during the multiplication and exodus stages, but absent during microcolony formation (Fig. 3B). Conversely, expression of *psm*β, an Agr-dependent gene, was observed only during maturation, within developing microcolonies (Fig. 3A). Agr-dependent virulence genes were more homogenously expressed across the cell population compared to the stochastic expression seen with Sae-dependent genes. The *nuc* reporter (shown in Fig. 2A) displayed both stochastic expression during early stages of development and homogenous expression within microcolonies, consistent with the observation that its expression is dependent on both Agr and Sae.

**FIG 4:**
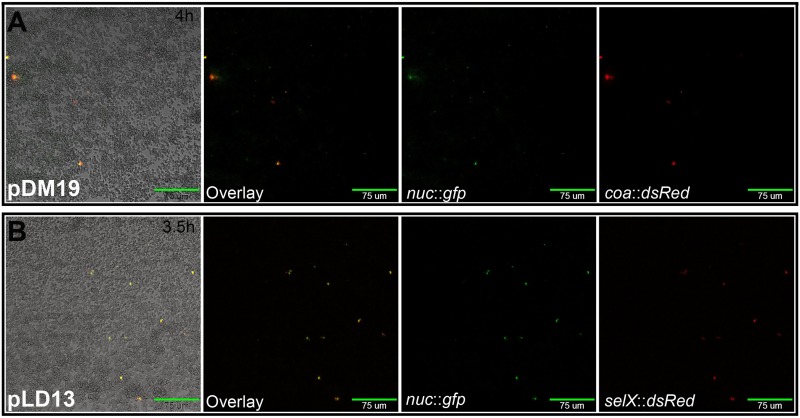
Coexpression of *nuc* and additional Sae-dependent genes during biofilm development. The images shown are of wild-type S. aureus cells harboring the dual reporter for (A) *nuc* and *coa* (pDM19) taken after 4 h of growth and (B) *nuc* and *selX* (pLD13) taken after 3.5 h of growth in a BioFlux system, where bright-field and epifluorescent images were acquired at 5-min intervals at ×200 magnification. Scale bars represent 75 μm. Overlay of FITC (GFP) and TRITC (DsRed) images shows overlap in the fluorescent signals indicating coexpression of these virulence factors within this subpopulation of cells. Images are representative of results from three independent experiments, each containing at least two technical replicates for each strain.

Given that stochastic expression of the Sae-regulated virulence genes was observed at the same stage of biofilm development, we asked whether these virulence genes were expressed within the same cells. To assess this, we generated dual-reporter constructs to enable observation of simultaneous expression of multiple genes of interest. In the experiment shown in Fig. 4A, the *nuc* promoter was fused to *gfp* and a second fluorescent reporter gene (*dsRed*) was used to generate a transcriptional reporter for the Sae-dependent gene, staphylocoagulase (*coa* [27]). Examination of this dual-reporter strain during biofilm development revealed that these two genes are coexpressed within the same subpopulation of cells. Similar results were observed with another dual-reporter construct containing a *nuc*/*selX* combination (Fig. 4B).

### Stochastic expression is also observed during planktonic growth.

**FIG 5:**
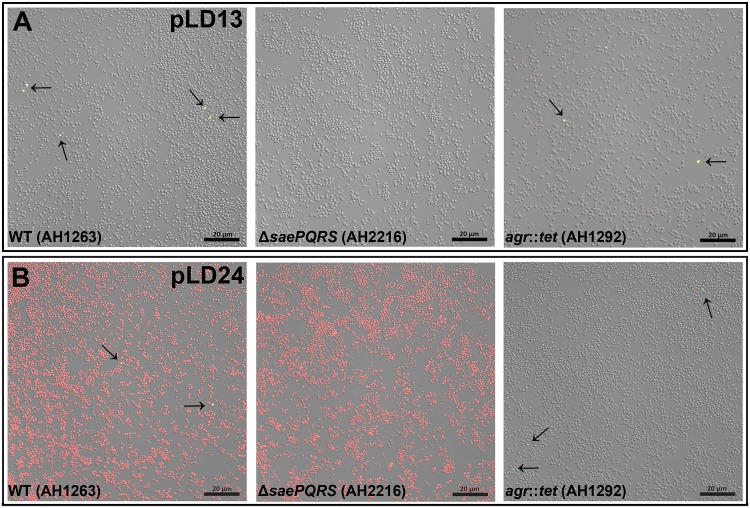
Sae- and Agr-dependent regulation of virulence genes during planktonic growth. The S. aureus wild-type strain (AH1263), *sae* mutant (AH2216), and *agr* mutant (AH1292) harboring dual gene expression reporters were grown aerobically with shaking in 3-ml glass culture tubes and imaged after cells had reached the stationary phase. (A) Cells harboring the *selX*::*dsRed*/*nuc*::*gfp* dual-reporter plasmid (pLD13). (B) Cells harboring the *psm*β::*dsRed*/*nuc*::*gfp* dual-reporter plasmid (pLD24). Images were obtained using confocal laser microscopy at ×630 magnification. The images shown are representative of results from two independent experiments and multiple fields of view observed during each experiment. Arrows highlight stochastic expression. Scale bars represent 20 μm.

As described above, we observed a subpopulation of high-expressing cells immediately after seeding of the biofilm experiments described for Fig. 1. As a result of this observation, we reasoned that stochastic expression of *nuc* (and of other Sae-regulated genes) would also be observed during planktonic growth. Thus, we grew several of our reporter strains to the stationary phase under standard planktonic conditions and examined them using confocal microscopy. As shown in Fig. 5A, stochastic expression was observed from our Sae-dependent dual-reporter construct, pLD13, indicating that *nuc* and *selX* are expressed in a subpopulation of cells during planktonic growth. Furthermore, stochastic expression of *nuc* and *selX* was also observed in the *agr* mutant but was absent in the *sae* mutant (Fig. 5A). Similarly to growth during biofilm development, stochastic expression was not observed with the Agr-dependent *psm*β reporter under planktonic conditions (Fig. 5B). Instead, expression of *psm*β (indicated by red fluorescence from the dual-reporter pLD24) was mostly homogenous across the planktonic wild-type population, with only slight cell-to-cell variation in signal intensity (Fig. 5B). Expression of *psm*β was unaffected by deletion of *saePQRS* but was absent in the *agr* mutant (Fig. 5B). In contrast, stochastic *nuc* expression was observed from the dual reporter (indicated by green fluorescence) in the wild-type and *agr* mutant strains but was absent in the *sae* mutant (Fig. 5B).

### Stochastic *nuc* expression and the exodus stage of development are dependent on *saeS*.

**FIG 6:**
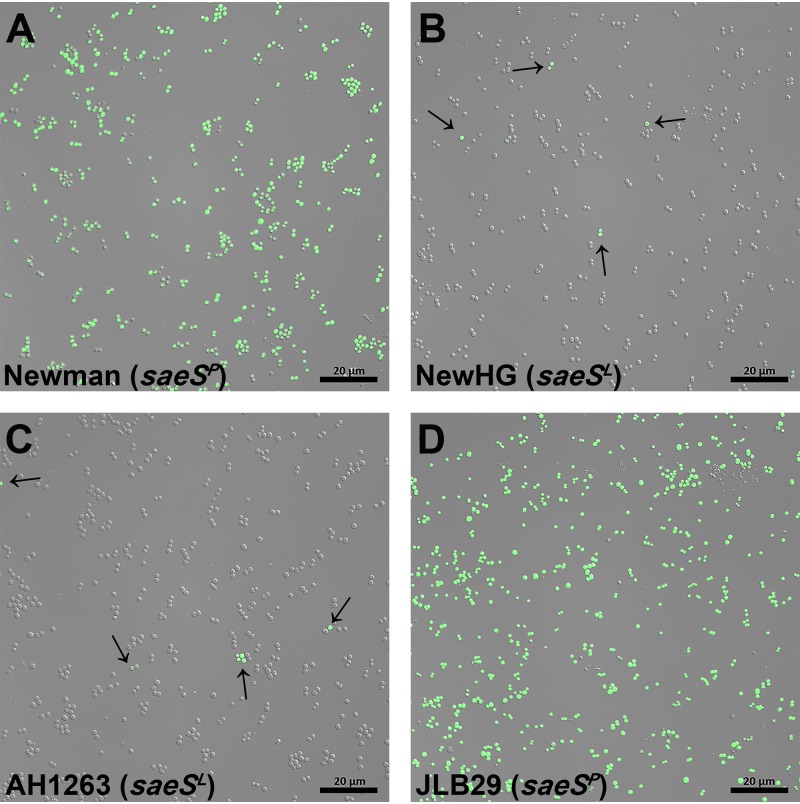
Effect of the *saeS^P^* allele on expression of *nuc*. S. aureus strains Newman (A), NewHG (B), AH1263 (C), and JLB29 (D) containing the *nuc*::*gfp* reporter plasmid (pCM20) were grown aerobically with shaking in 3-ml glass culture tubes. Bright-field and epifluorescent images were obtained using confocal laser microscopy at ×630 magnification. The images shown are representative of results from two independent experiments and multiple fields of view observed for all strains during each experiment. Images were acquired after approximately 12 h of growth. Arrows indicate stochastic expression. Scale bars represent 20 μm.

One exception to the stochastic virulence gene expression that we observed in the different strains tested was the S. aureus Newman strain, which demonstrated homogeneous expression of the *nuc*::*gfp* reporter across the entire population (Fig. 6A). This strain contains a natural variant of the *saeS* allele that encodes a SaeS variant in which amino acid 18 is changed from a leucine (SaeS^L^) to a proline (SaeS^P^), resulting in the constitutive kinase activity of SaeS and subsequent overexpression of Sae-regulated genes (28). Although it was presumed that this mutation resulted in an increase in the average expression levels of the virulence genes tested, the observation that the majority of the population expressed *nuc* suggests that the apparent increase in gene expression was actually a result of the loss of stochastic expression (an increase in the total number of cells within the population expressing virulence genes). To assess the potential impact of constitutive activation of SaeS on stochastic gene expression, the variant allele found in the Newman strain was introduced into the AH1263 background to generate strain JLB29 (29), followed by the transfer of the *nuc*::*gfp* promoter plasmid into this strain. As shown in Fig. 6D, the variant allele in JLB29 resulted in the conversion from stochastic *nuc* expression to homogeneous expression. In a reciprocal experiment, we monitored *nuc* expression in a Newman-derived strain (NewHG) in which the variant *saeS* allele was altered such that it produces the well-conserved SaeS^L^ protein (30). As shown in Fig. 6B, this change resulted in stochastic expression of the *nuc*::*gfp* reporter similar to that seen with the AH1263 strain (shown in Fig. 6C). Reporters for other Sae-dependent virulence factors such as *coa* (unpublished results) and *selX* (Fig. S4) showed the same results as were observed for *nuc*, with stochastic gene expression observed in the strains that harbor the *saeS^L^* allele but homogeneous expression in the strains containing the *saeS^P^* allele.

10.1128/mBio.03081-19.4 FIG S4 Stochastic expression of *nuc* and *selX* is dependent on SaeS activation in planktonic culture. S. aureus NewHG (A), Newman (B), AH1263 (C), and JLB29 (D) containing the *selX*::*dsRed*/*nuc*::*gfp* dual-reporter plasmid (pLD13) were grown aerobically with shaking in 3-ml glass culture tubes until the cells reached stationary phase. Images were obtained using confocal laser microscopy at ×630 magnification. The images shown are representative of results from two independent experiments and multiple fields of view. Arrows highlight stochastic expression. Scale bars represent 20 μm. Download FIG S4, TIF file, 4.7 MB. Copyright © 2020 DelMain et al. 2020 DelMain et al. This content is distributed under the terms of the Creative Commons Attribution 4.0 International license.

**FIG 7:**
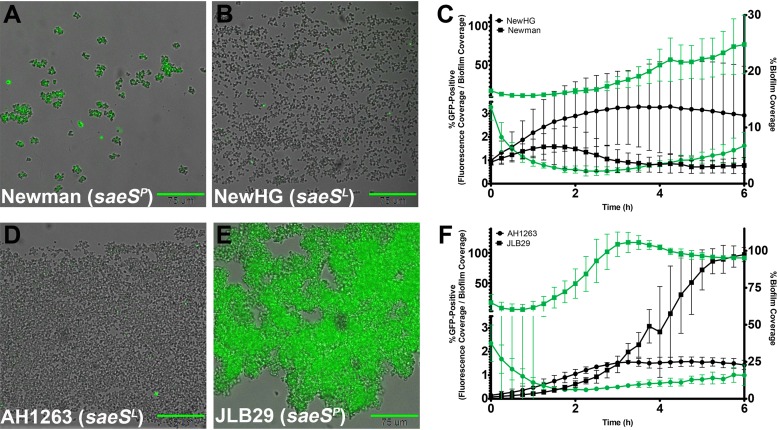
The *saeS^P^* allele affects stochastic expression and exodus during biofilm development. S. aureus strains containing the *nuc*::*gfp* reporter plasmid (pCM20) were grown in a BioFlux system, where bright-field and epifluorescent images were acquired at 5-min intervals at ×200 magnification. The biofilm images shown are overlays of bright-field and epifluorescent and are representative of results from three independent experiments, each containing at least two technical replicates. Images for NewHG (B), AH1263 (D), and JLB29 (E) were acquired at 4 h of growth, and the image for Newman (A) was acquired at 3 h of growth (nearly all cells were gone by 4 h). Scale bars represent 75 μm. Stochastic expression was observed in strains containing the SaeS^L^ allele (NewHG and AH1263, represented by circles in panels C and F, respectively). In the SaeS^P^ isogenic strains (Newman and JLB29, represented by squares in panels C and F, respectively), expression was mostly homogenous and occurred at a very high level. The plots (C and F) depict average percentages of channel area covered by cells (black symbols, plotted on the right *y* axis) and average percentages of biofilm-covered areas that are also GFP positive (green symbols, plotted on the left *y* axis) at 15-min intervals over a 6-h time period. The maximum values of the *y* axes were adjusted in some cases to ensure the visibility of the full range of mean values over the 6-h growing period. The data represent means of results from three independent experiments, each containing at least two technical replicates of each strain. Error bars represent the SD.

To determine the impact of the variant *saeS* allele on biofilm development, we grew the *saeS^L^* and *saeS^P^* strains in the BioFlux system. As was observed in planktonic culture, the biofilms produced by the *saeS^P^* strains (Newman and JLB29) were comprised almost entirely of *nuc*-expressing cells within 3 h of the start of the experiment (Fig. 7A and E). The biofilm phenotypes associated with these two strains were highly divergent both compared with each other and compared with their respective *saeS^L^* isogenic strains. JLB29 biofilms had a delayed multiplication stage, but accumulation occurred rapidly after 3 h (Fig. 7F). Ultimately, these biofilms failed to undergo exodus and formed dense cell aggregates that filled the channels by 6 h. Similarly to JLB29, Newman was slow to proliferate; however, unlike JLB29, this strain showed a reduced propensity to remain attached to the channel, as most cells were flushed from the surface within 3 h (Fig. 7C). These observations are consistent with previously published reports demonstrating the poor biofilm-forming ability of the Newman strain (31, 32). While NewHG biofilms (Fig. 7B and C) did not achieve the same level of channel coverage as those of AH1263 (Fig. 7D and F), this strain formed a more robust biofilm than strain Newman and exodus occurred shortly after the induction of the *nuc* reporter (Fig. 7C). Combined, the results from the planktonic and biofilm studies suggest that the SaeS protein plays an important role in mediating stochastic expression and biofilm development.

## DISCUSSION

The results of the current study reveal the ability of S. aureus to produce a specialized subpopulation of cells that specifically express virulence genes. These findings build on previous results demonstrating that a newly defined stage of biofilm development, designated “exodus,” is mediated by the stochastic expression of the *nuc* gene encoding staphylococcal nuclease (19). This secreted enzyme was shown to mediate the degradation of the extracellular DNA (eDNA) matrix of biofilm, resulting in the detachment of cells during the early stages of biofilm development and allowing microcolony formation. In contrast to the previous dogma, which was based on the presumption that all S. aureus cells within a population are similar with respect to virulence gene expression, our current results support a growing awareness that expression of some virulence genes is limited to a subpopulation of cells under standard planktonic growth conditions and during biofilm development (33, 34). We also demonstrated that the stochastic expression of these genes is likely to be mediated by the well-known SaePQRS regulatory system. In addition, our results reveal an intricate interplay between the Sae and Agr regulatory systems that coordinate virulence gene expression within the context of a developing biofilm.

Based on the effects of the Sae and Agr regulatory systems on expression of the different S. aureus virulence genes, we can divide these genes into three distinct regulatory groups: (i) virulence genes regulated by the Sae regulatory system, (ii) virulence genes regulated by the Agr regulatory system, and (iii) virulence genes regulated by both Sae and Agr. The results of the current study demonstrate that these three regulatory categories are neatly associated with different stages of biofilm development. For example, all the Sae-regulated genes tested exhibited stochastic expression during the early stages of biofilm development, while the Agr-regulated genes tested were all expressed during the maturation stage within developing microcolonies. Those virulence genes that are regulated by both Sae and Agr displayed both expression patterns. The results from the studies performed using our *nuc* reporters also demonstrate two distinct expression patterns whose differences were dependent on the growth conditions. Cells grown in planktonic culture demonstrated a clear pattern of Sae-dependent regulation that was independent of Agr. In contrast, under biofilm conditions, *nuc* expression was found to be Sae dependent during exodus but Agr dependent during maturation.

Although it seems clear that the Sae regulatory system is involved in the stochastic expression of the genes under its control, the molecular mechanism mediating this expression remains unknown. In general, stochastic expression is dependent on a so-called “bistable switch” that can dictate two different expression outcomes based on the achievement of a “threshold” level of transcription factors, usually involving an autoregulatory loop, that determines whether target genes are expressed or not. On the basis of the intrinsic “noise” generated in the transcription of genes (35, 36), the achievement of the threshold level of transcription of a bistable switch within a given cell determines whether the cell expresses a target gene (37). Classic examples of bistable switches are the natural competence (38–41) and sporulation (42–44) systems of Bacillus subtilis, the former causing approximately 15% of the population to become competent for natural transformation and the latter dividing the population into subpopulations consisting of sporulating cells and nonsporulating cells (44, 45).

The demonstration of a specialized subpopulation of cells dedicated to the expression of virulence genes is consistent with an emerging theme of virulence gene expression in S. aureus. Since the original discovery of the stochastic expression of *nuc* during biofilm development (19), similar observations of the bimodal regulation of other virulence genes have been reported. For example, García-Betancur et al. (33) have demonstrated that the Agr regulatory system plays an important role in the bifurcation of S. aureus into two distinct subpopulations, one that is destined for a biofilm lifestyle and the other for planktonic growth. Importantly, that study suggested that the Agr system itself functions as a bistable switch and that the magnesium concentration in different host niches influences the bimodal outcome. In another study, expression of the capsular polysaccharide operon was shown to be limited to a subpopulation of cells (34). Although *cap* expression was largely dependent on Agr, this regulatory system, as well as several others tested, was found not to be responsible for the stochastic control of this operon. Interestingly, *cap* expression was previously shown to be unaffected by the SaeS polymorphism found in Newman (46), suggesting there may be yet another regulatory strategy (distinct from Sae and Agr) involved in mediation of stochastic expression of the *cap* operon.

The N-terminal region of the SaeS histidine kinase component of the SaePQRS multicomponent regulatory system contains two transmembrane domains connected by a short linker peptide (28). This region is integral to sensing environmental stimuli and inducing an appropriate transcriptional response. Mutation of individual amino acids within the extracellular linker region of SaeS has previously been shown to disrupt the ability of this system to generate diverse responses in the presence or absence of stimuli (47). Furthermore, mutations to this region were shown to alter the autokinase and phosphotransferase activity rate of SaeS which corresponded to attenuated or enhanced virulence within a murine infection model (48). The results from the current study highlight how SaeS plays an important role in the stochastic expression of the target genes of the SaePQRS regulatory system by functioning as a molecular switch. A single point mutation found within the *saeS* gene of the Newman strain, well known for its ability to produce high levels of virulence factors (46), causes loss of stochastic expression, resulting in the entire cell population becoming fixed in an “ON” state (shown in Fig. 6 and 7). Introduction of this variant allele into AH1263 (JLB29) resulted in the conversion from stochastic *nuc* expression to homogenous expression within the population (Fig. 6D and 7E). Importantly, repair of the mutant allele in the Newman strain (NewHG) resulted in the restoration of stochastic expression, confirming the role of *saeS* in this mode of regulation (as indicated in Fig. 6B and 7B). Importantly, these findings also provide support for the model that the stochastic production of GFP within a subpopulation of cells is a consequence of genetic regulation and not an artifact of plasmid loss, growth rate, or oxygen concentration.

Interestingly, deletion of *saeP* (encoding a lipoprotein known to stimulate the phosphatase activity of SaeS) was shown to affect stochastic expression, as most cells within a *saeP* mutant biofilm express *nuc* (49). These results indicate that the mechanism controlling the bistable expression of virulence genes may involve specific interactions between the SaeP and SaeS proteins of this complex signal transduction system. Furthermore, recent studies of the role of CodY in the regulation of SaePQRS provided evidence that this regulatory protein also affects the stochastic expression of Sae targets such as *nuc* (7). This study demonstrated that a *codY* mutant harboring a *nuc*::*gfp* reporter displayed a significantly higher number of GFP-positive cells within the population than the wild-type parent strain, indicating that CodY contributes to stochastic expression of *nuc*, possibly via its role in the regulation of the *sae* P1 promoter, which drives expression of the components of the SaePQRS system. Ongoing studies are focused on defining the precise interactions between the Sae and CodY regulatory systems that mediate the bistable expression of virulence genes as well as on understanding the biological significance of this mode of regulation.

Overall, the results of this study reveal a complexity with respect to the regulation of virulence gene expression and biofilm development that had not been previously appreciated. Given the role of the Sae regulatory system in the stochastic control of virulence gene expression during biofilm development, it is possible that the biofilm mode of growth provides a better context to understand the regulatory dynamics that exist between the different S. aureus regulatory systems. Furthermore, analyses of cells grown in planktonic culture had been assumed to be relatively homogeneous, with, until recently, little consideration of differences between cells within the population. As such, many commonly used transcriptional profiling strategies had previously relied on the analysis of mRNA from entire cell communities, thus giving an average value of gene expression across the transcriptome and inadvertently neglecting biologically significant differences that exist within subpopulations of cells (50). We have learned a great deal from those studies, but it is becoming increasingly apparent that a full understanding of the complex interactions between the regulatory pathways may require consideration of the potential temporal expression patterns and regulatory checkpoints that are the hallmarks of development in more-complex multicellular organisms.

## MATERIALS AND METHODS

### Bacterial strains and growth conditions.

The S. aureus strains used in this study are described in Table 1. All S. aureus strains were grown in tryptic soy broth (TSB) (EMD Biosciences) or on TSB containing 1.5% agar (TSA). When appropriate, erythromycin (5 μg ml^−1^) and chloramphenicol (10 μg ml^−1^) were added to the growth media for plasmid selection and maintenance. Escherichia coli was grown in LB broth (Difco) or on LB containing 1.5% agar. All E. coli growth media were supplemented with ampicillin (100 μg ml^−1^) for plasmid selection. All experiments were started from fresh TSB overnight cultures supplemented with the appropriate antibiotic and grown at 37°C with shaking at 250 rpm.

**TABLE 1:** Bacterial strains and plasmids used for this study^*a*^

Strain or plasmid	Description	Reference or source
Strains		
DH5α	E. coli	
RN4220	Highly transformable restriction-deficient strain	
RN6390	USA300, CC8	
MW2	USA400, CC1	
SA564	USA100, CC5	
UAMS-1	USA200, CC30	
AH1263	USA300 CA-MRSA Erm^s^ LAC derivative lacking LAC-p03, CC8	47
AH1292	AH1263 a*gr*::tet	21
AH2216	AH1263 Δ*saePQRS*	47
JLB29	AH1263 with *saeS^P^* allele	29
AH3498	AH1263 Δ*saeP*	49
Newman	Commonly used laboratory strain, CC8	30
NewHG	Newman background with *saeS^L^* allele	30

Plasmids		
pCM20	*nuc* promoter::sGFP, Amp^r^ and Erm^r^	21
pCM11*plc*	*plc* promoter::sGFP, Amp^r^ and Erm^r^	This work
pCM11*sek*	*sek* promoter::sGFP, Amp^r^ and Erm^r^	This work
pCM11P2Agr	*agr* P2 promoter::sGFP, Amp^r^ and Erm^r^	This work
pMRSII	Dual-reporter shuttle vector, Amp^r^ and Cm^r^	This work
pLD9	*selX* promoter::sDsRed, Amp^r^ and Cm^r^	This work
pLD13	*selX* promoter::sDsRed, *nuc* promoter::sGFP, Amp^r^ and Cm^r^	This work
pLD24	*psm*β promoter::sDsRed, *nuc* promoter::sGFP, Amp^r^ and Cm^r^	This work
pDM19	*coa* promoter::sDsRed, *nuc* promoter::sGFP, Amp^r^ and Cm^r^	This work

a Amp, ampicillin; CA-MRSA, community-acquired S. aureus; Erm^s^, erythromycin sensitive.

### Construction of gene reporter plasmids.

For construction of gene reporter plasmids, PCR amplification of the promoter regions of *nuc*, *selX*, *sek*, *plc*, and *psm*β and the P2 promoter of *agr* was performed using genomic DNA isolated from AH1263 using primers Nuc1Green-F, Nuc1Green-R, pSelXRed-F, pSelXRed-R, pSEK-F, pSEK-R, pPCL-F, pPCL-R, PSMbetaRed-F, PSMbetaRed-R, pP2AGR-F, and pP2AGR-R (Table 2). The PCR products were purified using a DNA Clean & Concentrator kit (Zymo Research Corp.). For single gene reporter plasmids, purified PCR product was digested with HindIII and KpnI and ligated into pCM20 digested with the same enzymes. The sequences for these single reporters were confirmed using the sGFP-S-r primer (Table 2). For dual gene reporter plasmids, site-directed mutagenesis was used to eliminate the internal NdeI site in the sDsRed reporter gene of the pMRSI plasmid (51) for generation of the pMRSII plasmid using primers sDsRed-dNdeI-f and sDsRed-dNdeI-r. The purified PCR amplification product for *selX* and the pMRSII plasmid were digested with SpeI and BamHI, and then the digested products were ligated together to generate pLD9. Next, the purified PCR product for *nuc* was digested with KpnI and EcoRI and ligated into pMRSII digested with the same enzymes to generate pLD10. pLD10 and the purified PCR amplification product for *selX* were each digested with the restriction enzymes SpeI and BamHI and then ligated together to generate pLD13. pLD10 and the purified PCR amplification product for *psm*β were each digested with the restriction enzymes NheI and BamHI and then ligated together to generate pLD24. To generate pDM19, the *nuc* promoter region was first amplified from AH1263 chromosomal DNA using primers Dual-nuc-F and Dual-nuc-R (Table 2) and then digested with EcoRI and PstI and ligated into pSC14 (provided by the laboratory of Vinai Chittezham Thomas), which had been digested with the same enzymes to generate pDM17. Next, the *coa* promoter region was amplified from the AH1263 chromosome using the Dual-coa-F and Dual-coa-R primers (Table 2). PstI and BamHI was used to digest the *coa* amplification product as well as pDM17, and these two digest products were ligated together to generate pDM19 (Table 1). Sequencing was performed on dual reporters using primers sDsRed-S-r, sGFP-S-r, pCN51-S1-f, and pCN51-S-r (Table 2) to ensure that the appropriate dual reporters were generated. Plasmids were heat shocked into DH5α E. coli. As performed previously (19), the plasmids were purified using a Wizard Plus SV Miniprep DNA purification system (Promega Corporation) and transferred by electroporation into S. aureus strain RN4220. Transduction of the plasmids into AH1263 was performed using φ11 phage propagated on the plasmid-containing RN4220 strain.

**TABLE 2:** Primer sequences used for this study

Primer name	Sequence (5′→3′)
sDsRed-dNdeI-f	GTGAAGGTGAAGGACGTCTTTATGAAGGTACACAAACAG
sDsRed-dNdeI-r	CTGTTTGTGTACCTTCATAAGGACGTCCTTCACCTTCAC
pPlc-F	CCCAAGCTTATTCATTCACATTTTGGAG
pPlc-R	GCCGGTACCCTTTCTATATTTAATACATTAATTATACATC
pSek-F	CCCAAGCTTGGTAACTGCTCAAGAG
pSek-R	GCTGGTACCCCTTAAATTCTATTTATTCAG
pSelXRed-F	CCCACTAGTGTTGTCTCCTTTACTCCG
pSelXRed-R	CCCGGATCCCTTGATGTAAAGCTTTATTTGCTAC
Nuc1Green-F	CCCGGTACCAGTAAATTATAAGTTATACATCTCG
Nuc1Green-R	CCCGAATTCCTTTTTAGTTAATTTTAATATTAAACG
PSMbetaRed-F	CCCGCTAGCGTAATCACGG
PSMbetaRed-R	CCCGGATCCCTTAAAATTTAAATTTGAAG
pP2agr-F	CCCAAGCTTGTTCACTGTGTCGATAATCC
pP2agr-R	ACCGGTACCCCTCACTGTCATTATAC
sDsRed-S-r	CTGTTGATGGTTCCCAACCC
sGFP-S-r	GTAGCATCACCTTCACCCTCTC
pCN51-S1-f	CTCACATGTTCTTTCCTGCGTTATCC
pCN51-S-r	GTTCTTGTTGCTGTTCCTGTTCTG
Dual-nuc-F	GCCCGCTGCAGGTAAATTATAAGTTATACATCTCG
Dual-nucR	GCCGGAATTCCTTTTTAGTTAATTTTAATATTAAACG
Dual-coa-F	GCCGCTGCAGGTTTCGCTTTAGTCATTTGAT
Dual-coa-R	GCCGGGATCCATGTAATTGCCCAATCTACAT

### BioFlux 1000 biofilm assays.

A BioFlux 1000 microfluidic system (Fluxion Biosciences, Inc.) was used to assess biofilm development as previously described (19). BioFlux 1000 48-well plates were used for all experiments. Biofilm growth channels were primed by adding 200 μl of 50% TSB to the output wells and using a reverse flow for 5 min at 5.0 dynes/cm^2^. TSB in the output wells was replaced with 200 μl of fresh inoculum made from overnight-grown S. aureus cultures diluted to an optical density at 600 nm (OD_600_) of 0.8. A 300-μl volume of fresh TSB was added to the input wells. The growth channels were then seeded by applying a reverse flow for 2 s at 2.0 dynes/cm^2^. The seeded plate was left to incubate on the heated (37°C) stage of a BioFlux 1000 system for 1 h to allow cells to attach to the growing channel walls. Inoculum remaining in the input and the output wells was aspirated, and 1.3 ml of fresh 50% TSB was added to the input wells. A forward flow at 0.6 dynes/cm^2^ was then applied to the channels for 18 h. Bright-field and epifluorescence images were taken in 5-min intervals for a total of 217 time points. All epifluorescent images monitoring GFP expression were acquired using a fluorescein isothiocyanate (FITC) filter, and images representing DsRed expression were acquired using a tetramethylrhodamine (TRITC) filter. All biofilm experiments were repeated for a total of three independent experiments, each containing at least two technical replicates for each strain.

### Quantification of acquired biofilm assay images.

Images representative of the phenotypes recorded were observed and selected using BioFlux Montage software (Fluxion Biosciences, Inc.). Bright-field and epifluorescence images were calibrated to 0.323445 μm/pixel. For bright-field images, a threshold was set using the Threshold tool and Slider tool to include all cells within each image while excluding any background area. The total percentage of area covered within this threshold was designated the percentage of biofilm coverage, and these values were plotted over time. For epifluorescence images, the threshold was set to include all light areas (which were considered to represent fluorescing cells) and the total percentage of area covered within this threshold was designated the percentage of fluorescing cells. The area covered by fluorescing cells was divided by the total area covered by biofilm to produce the percentage of biofilm that was GFP positive. These values were also plotted over time. All time points were plotted in 15-min intervals using GraphPad Prism (GraphPad Software).

### Statistical analysis of biofilm quantifications.

Statistical analysis was performed by members of the College of Public Health Biostatistics Division at the University of Nebraska Medical Center as follows. PC SAS version 9.4 was used for all summaries and analyses. The SAS procedure GLIMMIX was used to fit B-splines for each of the outcomes (cell coverage and percentage of fluorescing cells) for each of the strains of interest. All possible pairwise comparisons were made at selected time points, and the *P* values were subjected to Holm simulation adjustment for multiplicity with a Holm-simulated stepdown procedure.

### Microscopy imaging of GFP- and DsRed-expressing reporters in planktonically grown S. aureus strains.

A 1-ml volume of fresh overnight cultures of S. aureus containing fluorescent reporter plasmids was centrifuged at 16,000 rpm for 5 min, and the pelleted cells were resuspended in 1× phosphate-buffered saline (PBS) to an optical density at 600 nm (OD_600_) of 8. A 5-μl volume of resuspended cells was added to a glass slide, and the glass slide was covered with a coverslip and sealed with clear nail polish. The samples were examined with a Zeiss LSM710 AxioObserver microscope (Zeiss Inc., Thornwood, NY). Cells were excited using 488-nm and 561-nm lasers for green and red fluorescent proteins, respectively. The images were acquired using a 63× oil objective. All planktonic culture experiments were repeated for a total of two independent experiments.
