# Supplementary figures and images for: Stochastic Expression of Sae-Dependent Virulence Genes during Staphylococcus aureus Biofilm Development Is Dependent on SaeS
Source: mBio. 2020 Jan 14;11(1):e03081-19. doi: 10.1128/mBio.03081-19 (PMC6960292; doi:10.1128/mBio.03081-19)

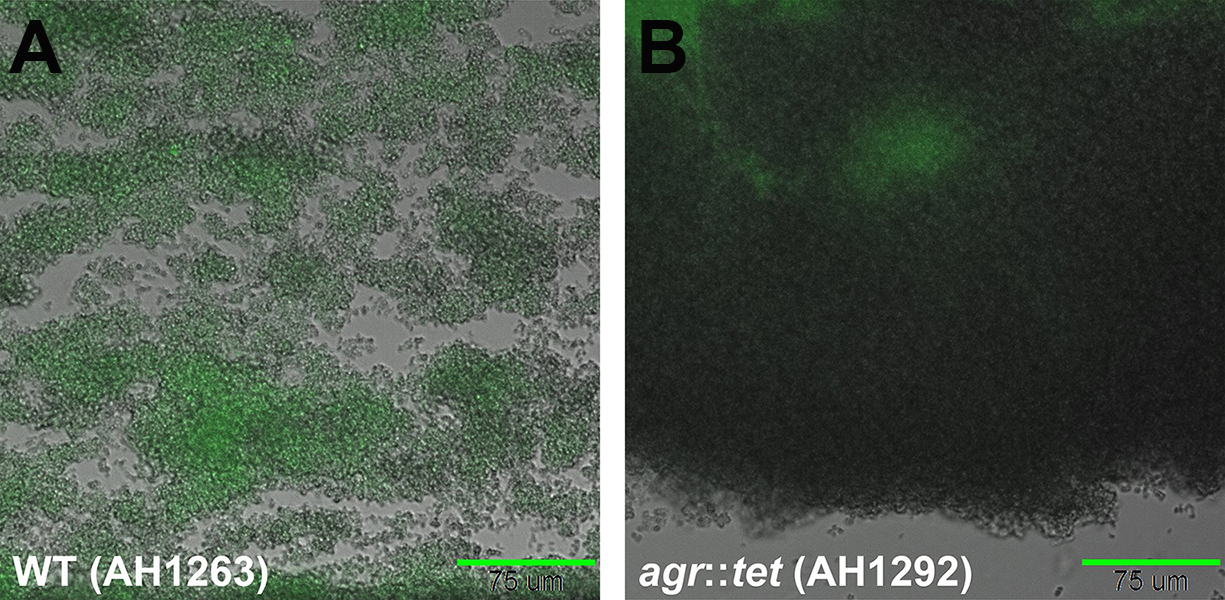

Supplement: FIG S1 [file mBio.03081-19-sf001.tif]

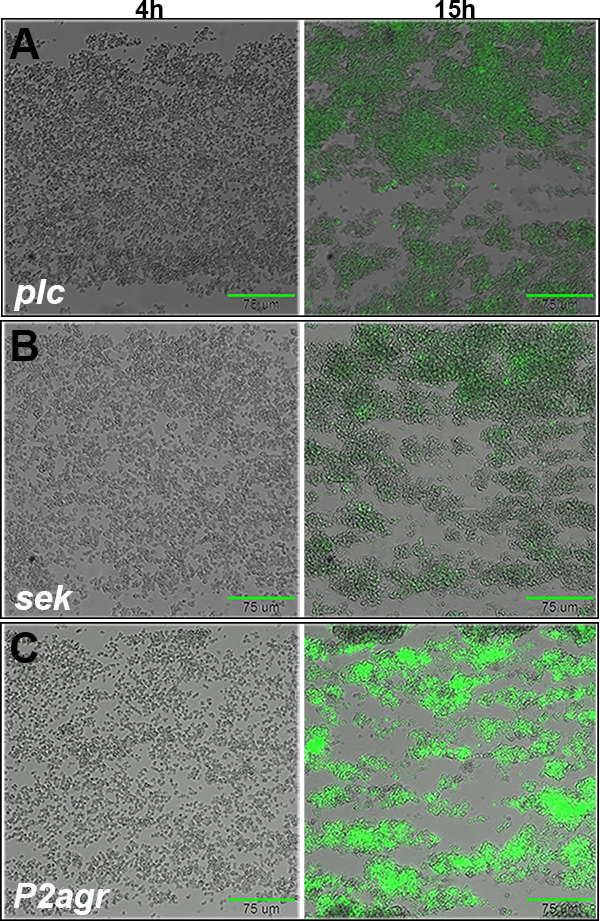

Supplement: FIG S2 [file mBio.03081-19-sf002.tif]

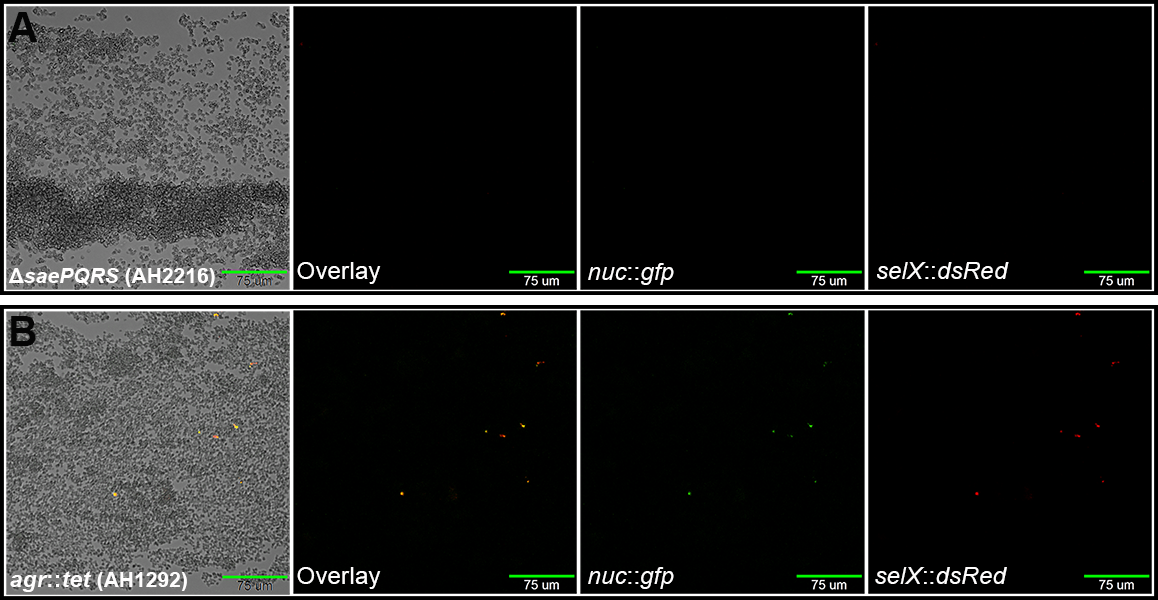

Supplement: FIG S3 [file mBio.03081-19-sf003.tif]

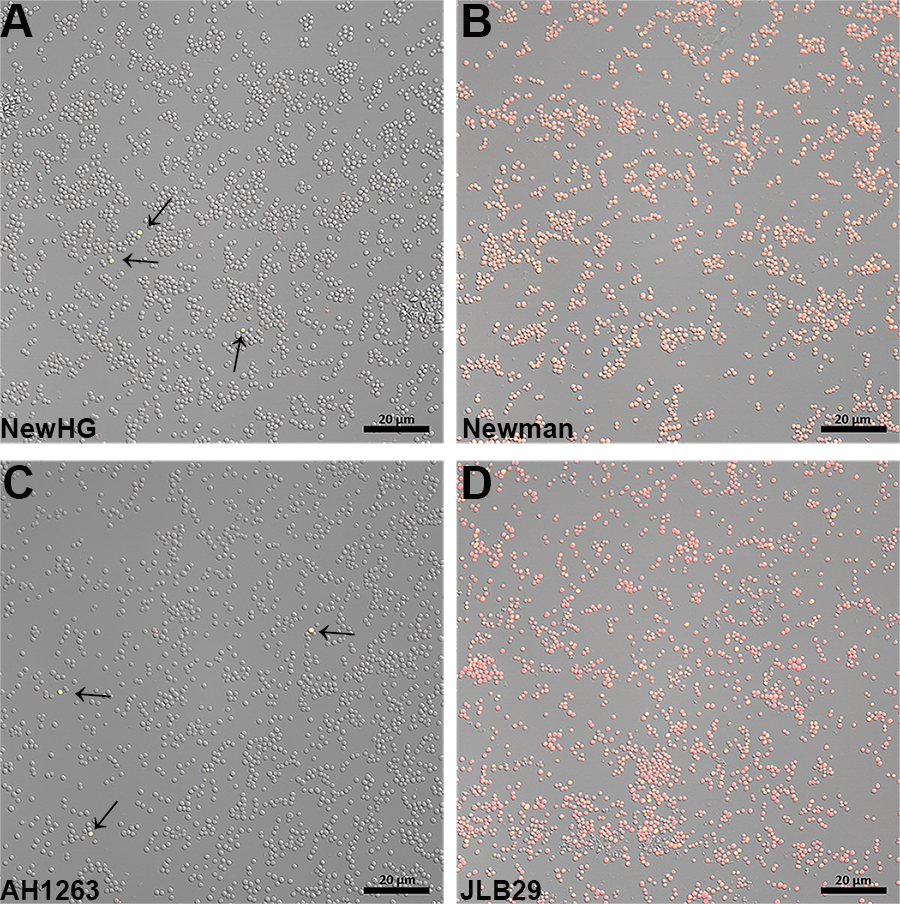

Supplement: FIG S4 [file mBio.03081-19-sf004.tif]
